# Culture of leukocyte-derived cells from human peripheral blood: Increased expression of pluripotent genes *OCT4, NANOG, SOX2*, self-renewal gene *TERT* and plasticity

**DOI:** 10.1097/MD.0000000000032746

**Published:** 2023-01-20

**Authors:** Yi-Jen Lee, Jehng-Kang Wang, Yu-Ming Pai, Alan Frost, Vip Viprakasit, Supachai Ekwattanakit, Hui-Chieh Chin, Jah-Yao Liu

**Affiliations:** a Department of Biochemistry, National Defense Medical Center, Taipei, Taiwan; b Autologous Stem Cell Technology Pty Ltd, Brisbane, Australia; c School of Veterinary Science, University of Queensland, Australia; d Department of Pediatrics and Thalassemia Center, Faculty of Medicine Siriraj Hospital, Mahidol University, Bangkok, Thailand; e Thalassemia Center, Faculty of Medicine Siriraj Hospital, Mahidol University, Bangkok, Thailand; f Department of Obstetrics and Gynecology, Tri-Service General Hospital, National Defense Medical Center, Taipei, Taiwan.

**Keywords:** leukocytes-derived, *NANOG*, *OCT4*, peripheral blood (PB), *SOX2*, *TERT*

## Abstract

There are few stem cells in human peripheral blood (PB). Increasing the population and plasticity of stem cells in PB and applying it to regenerative medicine require suitable culture methods. In this study, leukocyte populations 250 mL of PB were collected using a blood separator before that were cultured in optimal cell culture medium for 4 to 7 days. After culturing, stemness characteristics were analyzed, and red blood cells were removed from the cultured cells. In our results, stemness markers of the leukocyte populations Sca-1^+^ CD45^+^, CD117^+^ CD45^+^, and very small embryonic-like stem cells CD34^+^ Lin^−^ CD45^−^ and CXCR4^+^ Lin^−^ CD45^−^ were significantly increased. Furthermore, the expression of stem cell genes *OCT4 (POU5F1*), *NANOG, SOX2*, and the self-renewal gene *TERT* was analyzed by quantitative real-time polymerase chain reaction in these cells, and it showed a significant increase. These cells could be candidates for multi-potential cells and were further induced using trans-differentiation culture methods. These cells showed multiple differentiation potentials for osteocytes, nerve cells, cardiomyocytes, and hepatocytes. These results indicate that appropriate culture methods can be applied to increase expression of pluripotent genes and plasticity. Leukocytes of human PB can be induced to trans-differentiate into pluripotent potential cells, which will be an important breakthrough in regenerative medicine.

## 1. Introduction

Human peripheral blood (PB) contains a variety of stem cells, including mononuclear cells (MNCs) and hematopoietic stem/progenitor cells (HSPCs) from the bone marrow,^[1]^ mesenchymal stem cells (MSCs),^[2,3]^ endothelial progenitor cells (EPCs),^[4]^ and very small embryonic-like stem cells (VSELs).^[5,6]^ These cells express capacities of differentiation and regeneration with HSPCs/MNCs differentiating into hepatocyte,^[7]^ and MSCs exhibiting osteogenesis and adipose differentiation.^[2]^ EPCs can rejuvenate and repair endothelial cells of cardiovascular system,^[8,9]^ and VSELs generate non-hematopoietic progenitors of the 3 germ layers and cure damaged lung cells.^[10]^

HSPCs/MNCs, MSCs, EPCs express a variety of stem cell markers, including CD13, CD29, CD34, CD44, CD49e, CD54, CD71, CD73, CD90, CD105, CD106, CD117, CD133, CD166, and HLA-ABC.^[11]^ In addition, the stem cell marker Sca-1 is also expressed in thymic vessels, HSPCs,^[12]^ especially during liver differentiation of the embryo. HSCs differentiate into myeloid lineages and specific lymphoid subpopulations.^[13,14]^ For the regeneration function, the viability and repair of cardiomyocytes are related to the high population of Sca-1^+^ cells.^[14–16]^ Previous studies have reported the presence of rare VSELs stem cells in the PB.^[5,6]^ Animal experiments indicated that granulocyte colony-stimulating factor treatment can increase the ratio of CD34^+^ Lin^−^ CD45^−^, CD133^+^ Lin^−^ CD45^−^, and CXCR4^+^ Lin^−^ CD45^−^ cells of VSELs populations showing differentiation characteristics and pluripotency gene expression.^[5,6]^

Previous reported that the expression of 4 genes, *OCT3/4, SOX-2, Klf4*, and *c-Myc*, generates induced pluripotent stem cells that can differentiate into 3 germ layer cell types in vitro and teratoma.^[17]^ The CD34^+^ stem cells of umbilical cord blood with a high expression of *OCT4* and *SOX-2* genes also have the ability to differentiate into 3 germ layers.^[18]^ The MNCs of umbilical cord blood express the *Klf4* gene referred to as the neural differentiation potential.^[19]^ Recently, it was found that high expression of stem cell genes *OCT3/4, SOX-2, c-MYC, VIM, BMP4, NCAM*, and *BMPR2* in blood (*P* < .05) was also associated with anti-aging.^[20]^ The CD133^+^ Lin^−^ CD45^−^ cells of VSELs present in organ regeneration are also highly correlated with the expression of pluripotent genes *OCT4* and *NANOG*.^[21]^ These reports indicate that increased expression of pluripotent genes may have better self-renewal differentiation potential.

However, HSPCs/MNCs, MSCs, EPCs, and VSELs populations of PB in the steady state were very small, and pluripotent genes were also low.^[22]^ For proliferation of stem cell proliferation, the growth factor, which still has safety issues in clinical therapeutic applications, is often added during culture.

In this study, the leukocyte population of human PB was collected using a blood separator and cultured on free fetal bovine serum (FBS) and growth factors for 4 to 6 days by incubation at 5% CO_2_ and 37°C. After culture, the leukocyte-derived cells were named autologous multi-lineage potential cells (AMPC). The expression of stem cell markers of HSPCs/MNCs, MSCs, EPCs, and VSELs, 3 pluripotent genes (*OCT4, NANOG, SOX-2*), and self-renewal gene (*TERT*), and the differentiation ability of the 3 germ layers were investigated.

## 2. Materials and Methods

### 2.1. Isolation and culture

Informed consent was obtained from 74 healthy adult volunteers enrolled between August 2014 and August 2018, which were excluded chronic history, cancer, autoimmune defect, hepatitis B, hepatitis C, syphilis, and AIDS. In addition, determinate of complete blood count of volunteers follow reference ranges were essential that included white blood cells (neutrophils, lymphocytes, monocytes, eosinophils, and basophils), red blood cells, hemoglobin, hematocrit, and platelet count. PB was collected from healthy subjects aged 20 to 40 years by venesection into a 250 mL bag. For this study, basic information was summarized on Table 1 that included age and gender of healthy subjects of different experiments.

**Table 1:** Summary of basic information of volunteers.

Experiments	Age	Mean ± SD (age)	N	Male (N, ratio %)	Female (N, ratio %)
Flow cytometry	21–40	28.3 ± 4.4	63	41, 65.1%	22, 34.9%
qRT-PCR	24–34	28.0 ± 2.9	9	5, 55.6%	4, 44.4%
Differentiation	25–40	31.2 ± 6.1	14	6, 42.9%	8, 57.1%

qRT-PCR = quantitative real-time polymerase chain reaction, SD = standard deviation.

A concentrated leukocyte fraction was isolated from the sample using a Spectra Optia® apheresis system (CaridianBCT, Inc., Colorado). The isolated leukocyte fraction was then diluted in a mixture of albumin solution (Albuminar^R^-25, CSL Behring GmbH, Marburg, Germany) and low glucose DMEM medium (Gibco, Grand Land) at volume proportions of 60% (v/v) and 40% (v/v), respectively. Leukocyte cell suspension (30 mL) was then seeded in a 75 cm^2^ flask (CLS430641U, Corning) at 1 × 10^7^ cells/mL that was as day 0 sample autologously. The flask was incubated for 4 to 7 days at 37°C in a humidified incubator with 5% CO_2_ that was as day 4 to day 7 AMPC samples autologously.

NCCIT (Bioresource Collection and Research Center [BCRC] 60314) and SKOV-3 (HTB-77) cell lines were obtained from BCRC and American Type Culture Collection, maintained in RPMI medium (GIBCO) supplemented with 10% FBS. Cells were incubated at 37°C in a humidified incubator equilibrated with 5% CO_2_.

### 2.2. Flow cytometry analysis

Day 0 was compared with various culture days of AMPC autologously of the healthy volunteers on this study. Day 0 and day 4 or day 5 AMPC were harvested and washed with phosphate-buffered saline containing 2% FBS and centrifuged at 650 g at 4°C for 5 minutes, and the cell pellets were collected. The cell number was adjusted to 3 × 10^6^ cells per assay for flow cytometry analysis. FcR blocking reagent (Miltenyi Biotec B.V. & Co. KG, Bergisch Gladbach, Germany) was added and incubated at 4°C for 10 minutes. Fluorescence antibodies were used: anti-human CD13 PE, anti-human CD29 PE, anti-human CD38 PE, anti-human CD49e, anti-human CD54 PE, anti-human CD62L PE, anti-human CD73 PE, anti-human CD105 PE, anti-human HLA-DR PE, anti-human Lin APC, anti-human SSEA-1 PE, anti-human SSEA-3 PE, anti-human SSEA-4 PE, anti-human TRA-1-60 PE, and anti-human TRA-1-81 PE belonging to eBioscience brand (eBioscience. Inc. USA), 7-AAD, anti-human CD14 APC, anti-human CD31 APC/Cy7, anti-human CD45 FITC, anti-human CD71 APC/CY7, and anti-human CD184 APC/CY7 belonging to BioLegend brand (BioLegend Inc. San Diego), anti-human CD34 PE, and anti-human CD44 PE belonging to BD brand (bdbioscience.com., USA), which stood at 4°C for 60 minutes in the dark, then washed 3 times. Finally, 500 µL of staining buffer was added to the fluorescent antibody-labeled cells and kept at 4°C until analysis (BD FACSverse, bdbioscience.com., San Jose). Viable cells were identified using the FACSuite software (bdbioscience.com., San Jose), and the data are shown as logarithmic histograms. The experiment was repeated in triplicate.

### 2.3. Quantitative real-time polymerase chain reaction (qRT-PCR)

Day-0 and day-5 AMPC samples were individually collected from each volunteer, named sample 1 (S1), sample 2 (S2), sample 3 (S3), and sample 4 (S4). The S mean was S1, S2, S3, and S4 average. Each sample was individually cultured and analyzed for qRT-PCR experiments. qRT-PCR analysis was conducted on day 0 and day 5 AMPC with NCCIT and SKOV-3 as positive control groups. The ACTB gene was used as the reference gene. Day 0 (S1, S2, S3, and S4) and AMPC (S1, S2, S3, and S4) of samples were respectively as blank and treatment.

The red blood cells of day 0 (S1, S2, S3, and S4) and day 5 AMPC (S1, S2, S3, and S4) of samples were respectively lysed and extracted ribonucleic acid using 1 mL of TRIzol^R^ reagent (Invitrogen, Carlsband), and the products were immediately stored at −80°C.

Reverse transcription (RT) of 2 μg of total RNA was performed using Applied Biosystems™ High-Capacity cDNA Reverse Transcription Kit (Applied Biosystems, USA). The reaction conditions were set as follows: 25°C for 10 minutes, 37°C for 120 minutes, 85°C for 5 minutes, and 4°C.

20 μL RT product were diluted with 80 μL nuclease-free H_2_O for 5X-dilution RT product. A 10 μL reaction mixture was prepared, including 5.8 μL of SYBR Green (BioRad 1708880, BIO-RAD Laboratories, Inc), 2.2 μL of nuclease-free water, 1μL 5X-dilution RT product (20 ng), and 1μL forward and reverse primers (500 nM) (Table 2). Each sample was tested in triplicates. A Bio-Rad CFX Connect RT-PCR Detection System (BIO-RAD Laboratories, Inc. USA) was used with the following program: (95°C for 20 seconds), and 39 cycles (95°C, 5 seconds; 60°C, 30 seconds).

**Table 2:** Primer sequences to determine expression of stemness genes using quantitative real-time PCR.

Gene	Sequence (5’–3’)
*OCT4*	*F-GCCACACGTAGGTTCTTGAAT †R-TGATGTCCTGGGACTGGATTT
*SOX2*	*F -TGGCGA ACCATCTCTGTGGTC †R-ATTACCAACGGTGTCAACCTGC
*NANOG*	*F -CTTGGAAGCTGC TGGGGAAGG †R-TCACACGTCTTCAGGTTGCATGT
*TERT*	*F -TCAAGCTGACTCGACACCGT †R-TGGTCTTGAAGTCTGAGGGCA
*ACTB*	*F -CATGTACGTTGCTATCCA GGC †R- CTCCTTAATGTCACGCACGAT

* F = forward.

†R = reverse.

The BIO-RAD CFX Manager software (version 3.0, BIO-RAD Laboratories, Inc. USA) was used for the experimental setup and data analysis. The target genes were normalized to a reference gene. The ΔCq value and ΔΔCq were calculated for the fold determination of day 5 AMPC.

### 2.4. Osteogenic induction and staining

Day 4 AMPC 5 × 10^4^ cells were seeded in 12-well plates and cultured in induction medium or control medium on day 0 of the osteogenic differentiation period. Osteogenic induction medium consisted of low glucose DMEM, 1 μM dexamethasone (Sigma D4902, Sigma Chemical Co. Louis), 10 mM β-glycerophosphate (Sigma G9422), and 60 μM ascorbic acid 2-phosphate (Sigma A8960). The medium was changed every 4 days.

At day 18 of induction period, cells were fixed with 10% formalin solution then immersed in 2% (w/v) Alizarin Red S (ARS; Sigma A5533) staining solution (dissolved in distilled water, with pH adjusted to 4.2 with 0.1% NH_4_OH solvent) for 20 minutes at room temperature. Cells were observed and photos were taken using an inverted microscope (Olympus, CKX41, Olympus, Tokyo, Japan).

An alkaline dye mixture was prepared for alkaline phosphatase (ALP) staining (Supplemental Data S1, Supplemental Digital Content, http://links.lww.com/MD/I356). On day 12 of induction, AMPC were fixed with citrate, acetone, and formaldehyde (20%/ 50%/30%) and then stained with an alkaline dye mixture. Cells were observed and photos were taken using an inverted microscope (Olympus).

### 2.5. Neuron-like cells induction and immune-fluorescence staining

1.2 to 1.5 × 10^6^ day 4-AMPC was seeded in a fibronectin (10 μg/ mL)-coated 24-well plate supplemented with a basal medium containing 30% FBS and low glucose DMEM (Gibco, Grand Land) medium on day 0. When cells were adherent on day 4, a co-culture system was established by 0.4 μm trans-wells (Costar 3470, CORING CO) in a 24-well plate. The upper deck (trans-wells) was seeded with 7 × 10^4^ PC-12 (BCRC 60048) cells to induce neuronal differentiation of AMPC on the lower deck (24-well plate). PC-12 was changed every 3 days.

PC-12 is a rat adrenal pheochromocytoma that synthesizes and stores the catecholamine neurotransmitters dopamine and norepinephrine.^[23]^

On day 19, immunocytochemistry fluorescence staining was performed on the cells. The cells were fixed, blocked, and labeled with antibodies (Supplemental Data S3, Supplemental Digital Content, http://links.lww.com/MD/I358). Nestin and neurogenin 3 proteins and DAPI expression were observed using a fluorescence microscope (Olympus, CKX41, fluorescence: CKX-RFA).

### 2.6. Cardio-like cells induction and immune-fluorescence staining

1.2 to 1.5 × 10^6^ day 4-AMPC was seeded in a fibronectin (10 μg/ mL) coated 24-well plate on day 0 and supplemented with a basal medium for 10 days (replaced every 3 days).

From day 11 to day 29 the basal medium was replaced with the cardiomyogenic induction medium consisting of the basal medium, 2% insulin (Gibco™ 12585014, Life Technologies Co., USA), 10 ng/mL EGF (eBioscience), and 10 ng/mL β−FGF (eBioscience). The induction medium was replaced every 3 days.

On day 29, the cells were fixed, blocked, and labeled with antibodies (Supplemental Data S3, Supplemental Digital Content, http://links.lww.com/MD/I358). Myogenin, troponin I, and α-actinin proteins and DAPI expression were observed using a fluorescence microscope.

### 2.7. Hepatocyte-like cells induction and immune-fluorescence staining of AMPC

1.5 to 2 × 10^6^ day 4-AMPC was seeded in a fibronectin (10 μg/ mL) coated 24-well plate on day 0 and supplemented with a basal medium. The cells were maintained on basal medium until day 10 the medium was replaced every 3 days.

From day 11 to day 22 the basal medium was replaced with a neo-hepatocyte induction medium consisting of basal medium, 1 μM dexamethasone, 20 ng/mL human HGF (eBioscience), and 10 ng/mL human β−FGF.

On day 22, the cells were fixed, blocked, and labeled with antibodies (Supplemental Data S3, Supplemental Digital Content, http://links.lww.com/MD/I358). Connexin 32, cytochrome P450 (CYP1A1) and albumin proteins and DAPI were observed using a fluorescence microscope.

### 2.8. Statistical analyses

All data are shown as mean ± SEM from 3 independent experiments. The statistical significance of the data was determined using Student *t* test, *P* values <.05 were recognized as significant and marked with “*.”

## 3. Results

### 3.1. AMPC exhibit HSPCs/MNCs, MSCs, EPCs, HSCs and VSELs stem cells potentials

**Figure 1.**
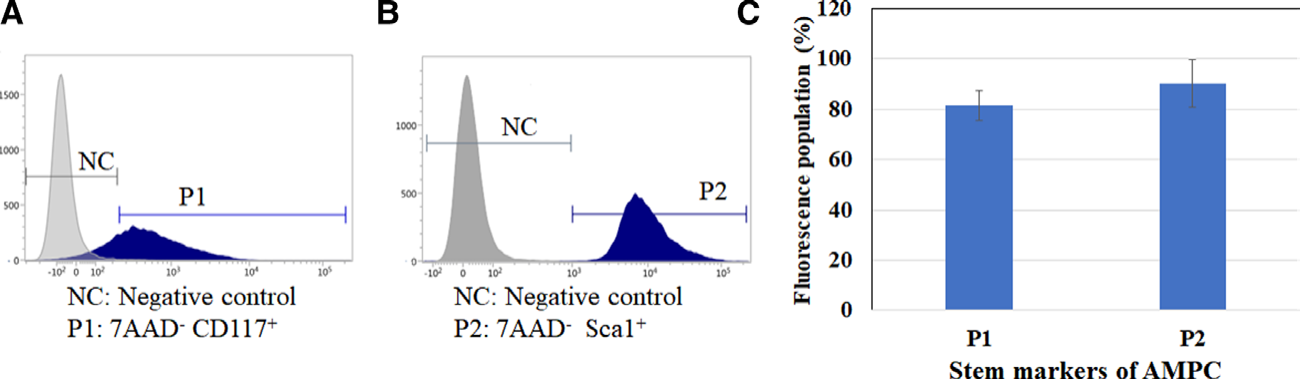
Enrich stem markers CD117 and Sca-1 expression in AMPC. AMPC samples were obtained and collected using CD45 MicroBeads (MACS) on Day 4. Fluorescently determined expression of these cells was analyzed by flow cytometry, including non-fluorescence-stained AMPC cells as a negative control (NC), (A) 7-AAD^−^ CD117^+^ (P1) cells, and (B) 7-AAD^−^ Sca-1^+^ (P2) cells. (C) Populations of P1 and P2, which were determined via flow cytometry with PECy7- and APCCy7-conjugated antibodies against CD117 and Sca-1, respectively; AMPC (N > 3) was analyzed. AMPC = autologous multi-lineage potential cells.

The expression of CD13, CD14, CD29, CD31, CD44, CD49e, CD54, CD62L, CD73, CD105, and HLA-DR on AMPC was related to some HSPC/MNC, MSCs, and EPCs characteristics (Table 3). The CD29 and CD44 markers showed a high ratio of 90% to 100% on AMPC. The CD34 and CD38 belong to HSCs markers which, respectively, showed 2% to 67% and 37% to 87% on AMPC (Table 3). Furthermore, AMPC also expressed high ratios of CD117 and sca-1 at 81.37% ± 5.81% and 90.23% ± 9.53%, respectively (Fig. 1).

**Table 3:** CD marker analysis of AMPC by flow cytometry.

Markers	AMPC (%)*
CD13	**2–70**
CD14	**3–46**
CD29	**90–100**
CD31	**47–94**
CD34	**2–67**
CD38	**37–83**
CD44	**90–100**
CD49e	**63–89**
CD54	**50–73**
CD62L	**4–88**
CD73	**5–70**
CD105	**9–89**
HLA-DR	**11–86**

AMPC = autologous multi-lineage potential cells.

* AMPC (%): Determine by flow cytometry analyzed results, was calculated by [(indicated CD markers-fluorescence cell numbers of AMPC) ÷ (total cell numbers of AMPC)] × 100%.

**Figure 2.**
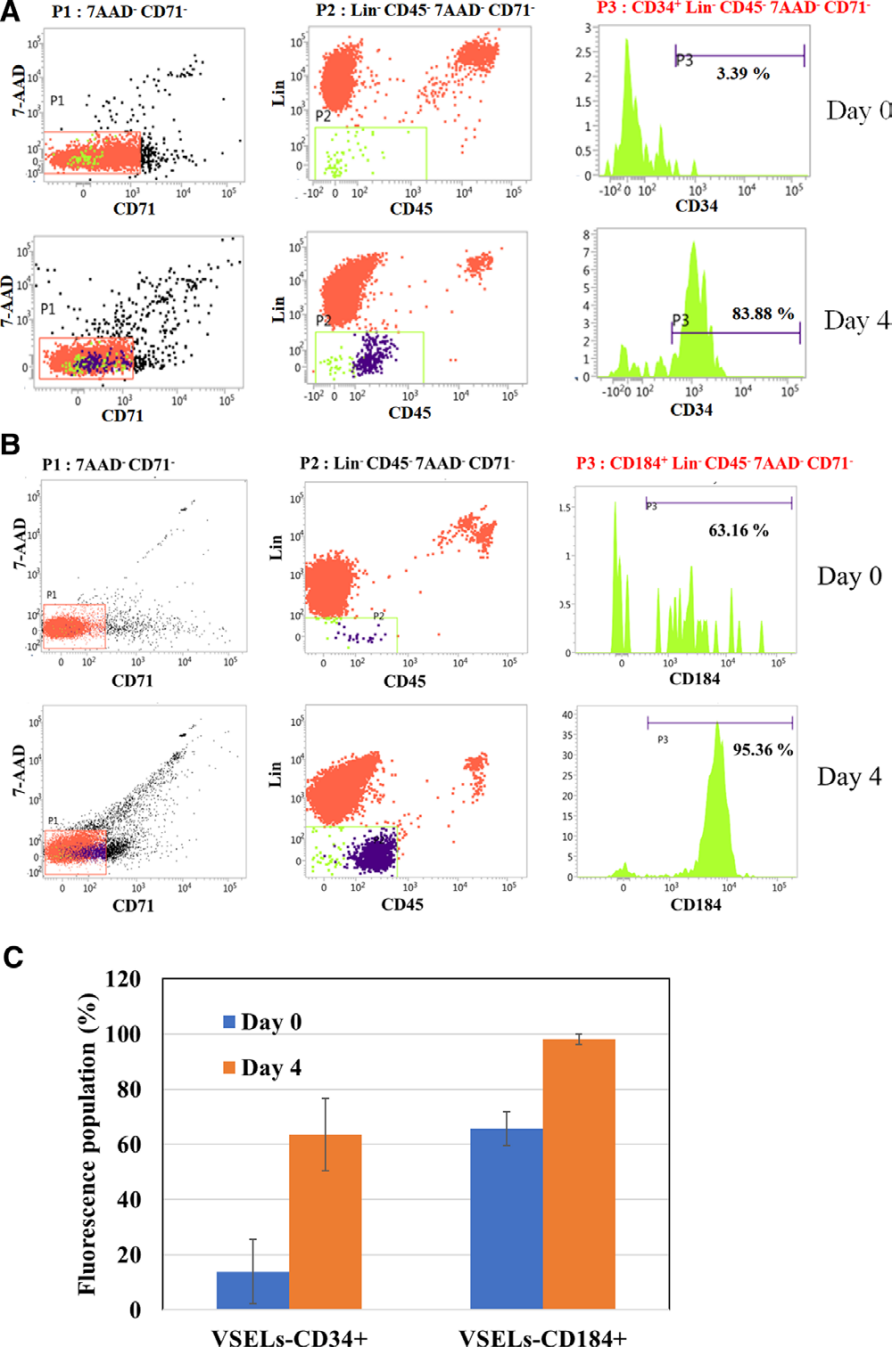
Enrich VSELs markers CD34 and CD184 expression in AMPC. The AMPC samples were collected on days 0 and 4. Fluorescently determined expression in these cells was analyzed using flow cytometry. (A) P1: 7AAD^−^ CD71^−^ cells, P2: Lin^−^ CD45^−^ CD71^−^ cells, and P3: CD34^+^ Lin^−^ CD45^−^ CD71^−^ cells. (B) P1: 7AAD^−^ CD71^−^ cells, P2: Lin^−^ CD45^−^ CD71^−^ cells, and P3: CD184^+^ Lin^−^ CD45^−^ CD71^−^ cells. (C) Fluorescence populations of VSELs-CD34 + and VSEL-CD184 + that were determined via flow cytometry with the indicated antibodies; AMPC (N > 3) were analyzed. AMPC = autologous multi-lineage potential cells, VSELs = very small embryonic-like stem cells.

Our results indicated that the CD34^+^ Lin^−^ CD45^−^ 7AAD^−^ CD71^−^ (VSELs-CD34^+^) (Fig. 2A) and CD184^+^ Lin^−^ CD45^−^ 7AAD^−^ CD71^−^ (VSELs-CD184^+^) cells (Fig. 2B) of VSELs were enriched compared with day 0, with AMPC expressing high ratios on day 4 of 63.51% ± 13.12% and 98.02% ± 1.89%, respectively (Fig. 2C).

### 3.2. AMPC showed OCT4, SOX2, and NANOG and TERT stemness genes expression

**Figure 3.**
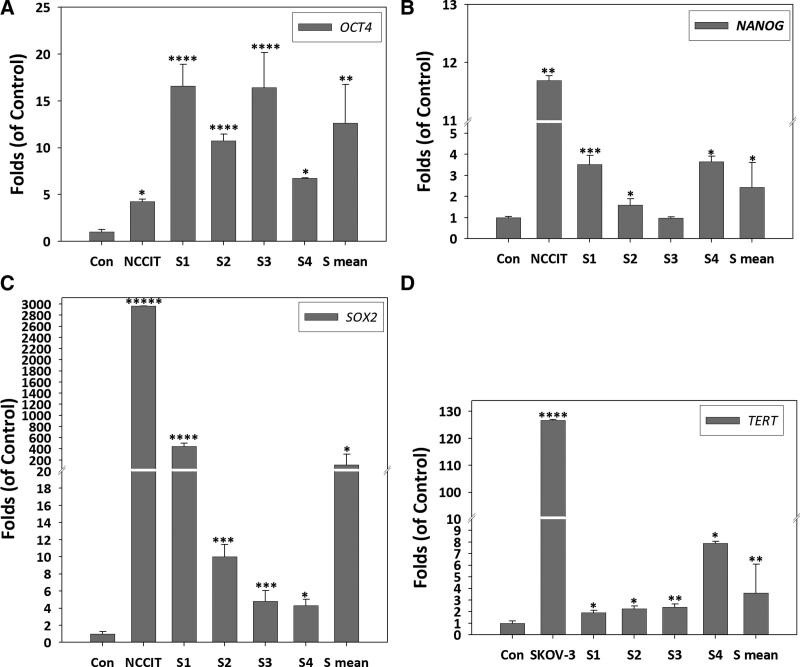
AMPC exhibit stemness and self-renewal characteristics. (A–C) Stemness genes *OCT4, SOX2*, and *NANOG* of NCCIT control (Con.) and AMPC samples S1, S2, S3, S4 and corresponding average (S mean) expression by qRT-PCR analysis. Each sample was tested in triplicate. (D) Self-renewal gene *TERT* gene expression of Con., SKOV3 and AMPC samples S1, S2, S3, S4 and the corresponding average by qRT-PCR analysis. Each sample was tested in triplicate. Target gene of qRT-PCR data were normalized to a reference gene *ACTB* on each. (*t* test, *: *P* < .05, **: *P* < .005, ***: *P* < .0005, ****: *P* < .00005, *****: *P* < .000005). AMPC = autologous multi-lineage potential cells, qRT-PCR = quantitative real-time polymerase chain reaction.

qRT-PCR analysis of the stemness genes *OCT4, SOX2, NANOG* and *TERT* showed changes in gene expression in cells during the culture period between day 0 and day 5. S1, S2, S3, and S4 samples were taken from day 5 of AMPC, day 0 (control), and NCCIT (positive control). The S mean (S1, S2, S3, and S4 average) fold changes in *OCT4, SOX2*, and *NANOG* showed, 12.62-fold (*P* < .005), 116.72-fold (*P* < .05), and 2.43-fold (*P* < .05) upregulation of gene expression, respectively, on day 5 (Fig. 3A–C). *TERT* gene expression showed a 3.61-fold (*P* < .005) increase on day 5 (Fig. 3D). Interestingly, we observed that *OCT4, SOX2, NANOG*, and *TERT* genes exhibited different expression profiles between S1, S2, S3, and S4 donors, suggesting that although leukocytes acquired stemness after culture, the effect varied among biological replicates.

### 3.3. Embryonic marker less expression in AMPC

**Figure 4.**
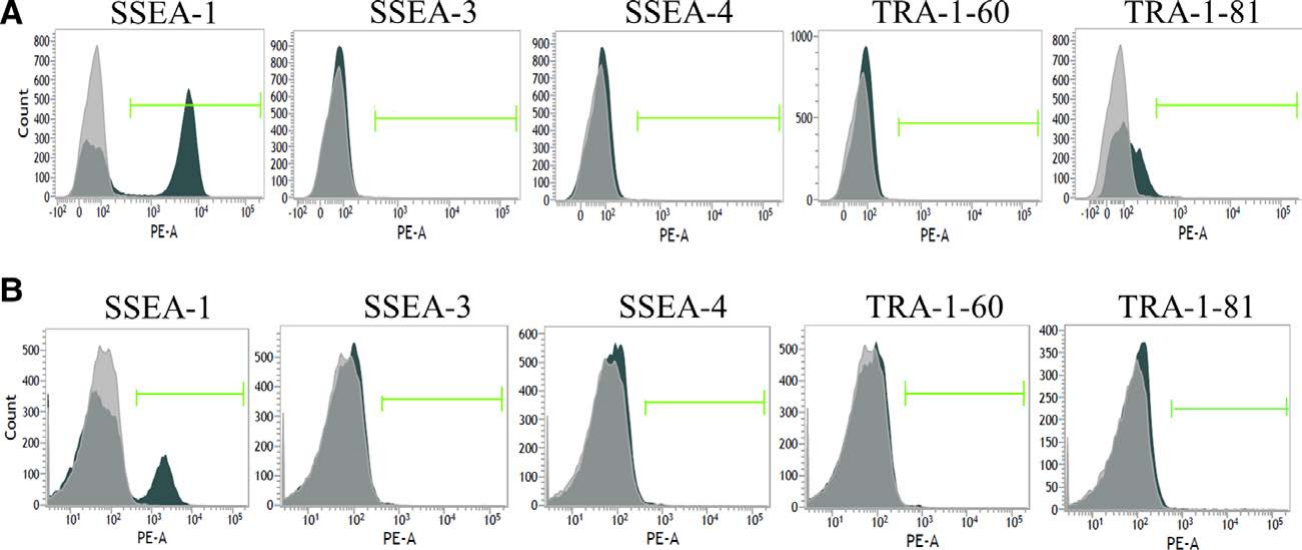
AMPC embryonic marker expression. Flow cytometry data showing the expression of SSEA-1, SSEA-3, SSEA-4, TRA-1-60, and TRA-1-81 in AMPC. SSEA-1, SSEA-3, SSEA-4, TRA-1-60, and TRA-1-81 expression was analyzed by FACSVerse (BD) in (A) A0 (day-0) cells (B) and AMPC on day 5. Fluorescent cells were quantified using FACSuite software using unstained expression as a fluorescence correlation. AMPC = autologous multi-lineage potential cells.

Embryonic markers SSEA-1, SSEA-3, SSEA-4, TRA-1-60, and TRA-1-81 of leukocytes and AMPC expression were analyzed by flow cytometry on both day 0 and day 5 of culture (Fig. 4A and B). Day 5 AMPC expressions of SSEA-3, SSEA-4, TRA-1-60, and TRA-1-81 were 0.30% ± 0.19%, 0.40% ± 0.33%, 0.34% ± 0.22%, and 0.33% ± 0.46%, respectively. SSEA-1 decreased expression from 50.94% ± 6.03% before to 17.61% ± 3.13% after culture.

### 3.4. Induction of osteoblasts from AMPC

**Figure 5.**
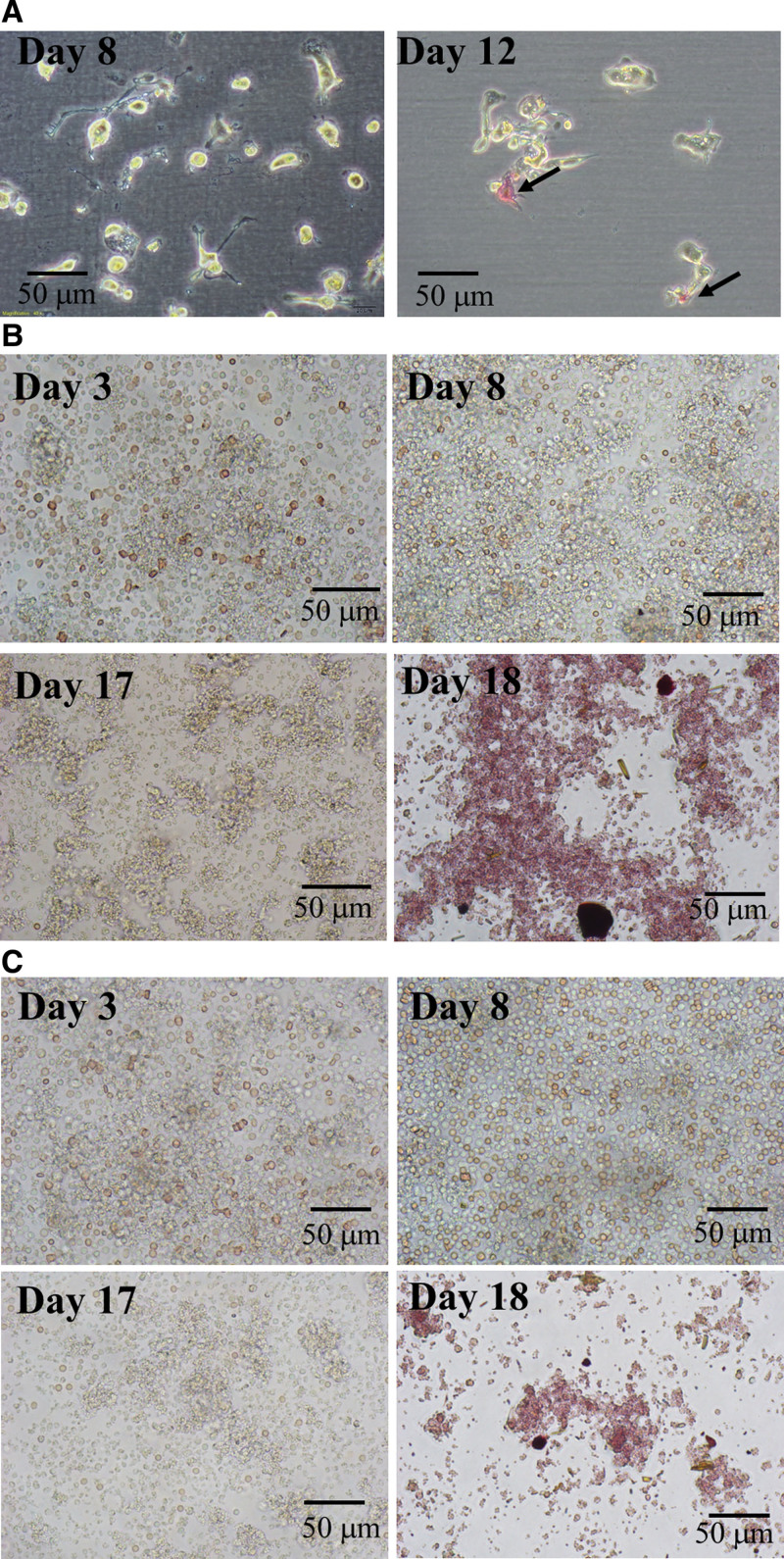
AMPC exhibit osteogenic differentiation. (A) ALP expression of AMPC. Osteogenic differentiation of AMPC after induction shown by alkaline phosphatase expression. The alkaline mixture staining was indicated by black arrows on the day 12. (B) Calcium accumulation expression of AMPC. Calcium accumulation by ARS staining in osteogenic differentiation of AMPC after 17 days induction and (C) uninduced AMPC. ALP = alkaline phosphatase, AMPC = autologous multi-lineage potential cells, ARS = Alizarin red S.

Bone mineralization is an essential process of osteoblasts, ALP and ARS are highly expressed in the cells of mineralized tissue and plays critical indications.^[24]^ AMPC was obtained after 12 days of induction and fixed in citrate–acetone-formaldehyde solution, then stained with an alkaline dye mixture (Supplemental data S1, Supplemental Digital Content, http://links.lww.com/MD/I356). For ALP expression, differentiation progress was observed on day 8 and day 12 using an inverted microscope (OLYMPUS). On day 12, inducted AMPC shown ALP expression (Fig. 5A).

The mineralization phase commenced from day 3 of induction in AMPC, and the cells were found to flat progressively on day 8. The morphology of differentiated AMPC cells was observed after 17 days of osteogenic induction. On day 18, differentiated AMPC showed abundant calcium accumulation as determined by ARS staining (Fig. 5B). Specially, AMPC shown mineralization on day 12 and day 17 of induction. Interestingly, the uninduced control group of AMPC also showed some calcium accumulation by ARS staining (Fig. 5C).

### 3.5. Induction of neurons, cardiomyocytes, and neo-hepatocytes from AMPC

**Figure 6.**
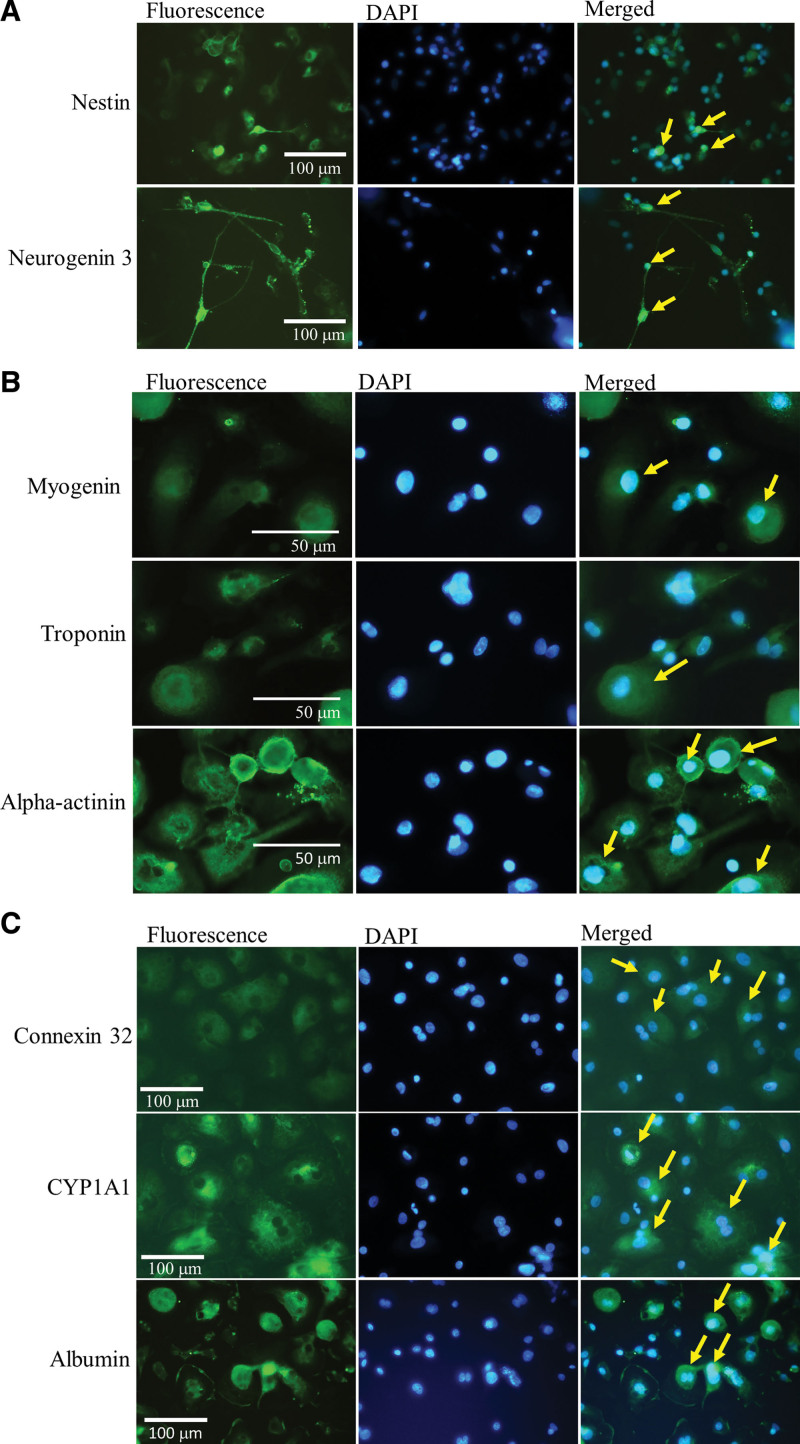
AMPC exhibit neuron-like, cardio-like, and hepatocyte-like differentiated potentials. (A) Induced AMPC expressed neural progenitor markers nestin and neurogenin 3 on day 19 during the PC-12 cells co-culture period. (B) Induced AMPC show cardiomyogenic protein expression, myogenin, troponin I, and alpha-actinin on day 29. (C) Induced AMPC show hepatocyte protein expression, connexin 32, CYP1A1 and albumin on day-22. Merged FITC-DAPI images of target proteins of neuron-like, cardio-like, and hepatocyte-like were indicated by yellow arrows. AMPC = autologous multi-lineage potential cells.

AMPC were co-cultured with PC-12 cells during the neuronal induction period. The differentiated cells displayed a multipolar elongated morphology with marked neural protein expression and displayed a specific neural multipolar morphology on day 21 like those of neurons and astrocytes. The expression of neuronal progenitor markers, including nestin and neurogenin 3 proteins, was observed on day 21 (Fig. 6A).

After 19 days of cardiomyocyte induction, differentiated AMPC showed expression of myogenin, troponin I and α-actinin proteins (Fig. 6B) that were localized in the cytoplasm of adherent cells.

Under neo-hepatocyte induction conditions, AMPC’s morphology of AMPCs became larger and flatter with fibroblast-like morphologies on day 22. The induced cells showed connexin 32, CYP1A1 and albumin proteins expression on day 22 (Fig. 6C).

Our results indicated that AMPC expressed neurons, cardiomyocytes, and neo-hepatocyte differentiation potentials after optimal induction culture periods.

## 4. Discussion

AMPC were isolated and cultured from human PB, which is a heterogeneous pool of cells with several cell types that show some stem markers of HSPCs/MNCs, MSCs, EPCs, and VSELs. We suggest 2 major reasons for the difference in expression between AMPC and other HSPCs/MNCs, MSCs, EPCs, HSCs,^[24,25]^ and VSELs^[5,10,26,27]^ stem cells, including the addition of growth factors for culture and granulocyte colony-stimulating factor treatment for enriched stem cell collection for other stem cells,^[5,28]^ but not for AMPC. Our results indicated that CD29 and CD44 achieved 90% to 100% expression ratios in AMPC (Table 3). CD29 expression can increase upon activation of CD4^+^ T cells and CD8^+^ T cells, which efficiently produce pro-inflammatory cytokines interferon-γ, interleukin-2, and tumor necrosis factor-α and cytotoxic molecules. High CD29 expression indicates effective therapeutic T-cell products.^[29,30]^ Previous experiments on animals have shown that CD44 deficiency is related to reduce leukocyte recruitment and delayed healing of wounds.^[31]^ Increasing CD44 expression can increase leukocyte adhesion to inflamed sites for therapeutic purposes.^[32]^ In AMPC, high expression of CD29 and CD44 helps in the homing of stem cells for the repair and regeneration of autologous therapy.

In this study, AMPC upregulated the expression of the classical stemness- and pluripotency-related genes *OCT4, SOX2*, and *NANOG*.^[33]^ The expression of these 3 genes is attributed to pluripotency in stem cells and the maintenance of stemness, and is a molecular hallmark of pluripotency.^[34]^ The *TERT* gene is involved in self-renewal in stem and progenitor cells.^[35]^ Its overexpression also indicates pluripotency and stemness, as upregulation is proportional to increased telomerase activity, which maintains the undifferentiated state of stem cells.^[36]^

The gene expression changes in AMPC during cell culture (day 0–day 6) showed upregulation of the pluripotency genes *OCT4, SOX2*, and *NANOG* (Supplemental data S2A–C, Supplemental Digital Content, http://links.lww.com/MD/I357). Similarly, increased *TERT* was observed by qRT-PCR. These results show the gradual upregulation of these genes, peaking between days 2 and 4 of culture (Supplemental Data S2D, Supplemental Digital Content, http://links.lww.com/MD/I357).

Varying intensities of gene expression were observed between S1, S2, S3, and S4 samples obtained from different blood donors, which could be attributed to the biological variation among individuals. Interestingly, the downregulation of these genes was also observed in some samples after a peak in gene expression. Cumulative studies on the downregulation of these genes indicate an early transitional period in which cells begin to differentiate.^[37–39]^ Summary our results, we found genes *OCT4, SOX2, NANOG*, and *TERT* expression were different uptrends and crests between day 0 and day 7 on each sample. We preliminarily concluded genes *OCT4, SOX2, NANOG*, and *TERT* of PB of each sample were found to express different uptrends and increasing ratio compared with autologous day-0 sample (before culture) by isolation and cultivation methods. These observations suggest that AMPC differentiation is associated with stem cell gene expression.

For AMPC’s regeneration and repair applications, was further verified by its potential to differentiate into cells of various germ layer lineages and its relation to optimal induction culture medium, including neo-hepatocyte-like, neuron-like, osteoblast-like, and cardiac-like cells. In our study results, AMPC there were functional markers expressions after induction culture of various differentiated germ layer lineages. Such as cardiac cells, α-actinin (Fig. 6B) is an actin-binding and microfilament protein that provide as a important component of template for the fusion of Z-disk precursors, Z bodies, and subsequent striation.^[40]^ Mature hepatocytes exhibit functions including albumin production, glycogen storage, drug transporter activation, and cytochrome P450 (CYP family) activity expression.^[41]^ In neo-hepatocyte differentiation of AMPC experiments, shown connexin 32, CYP1A1 and albumin proteins expression (Fig. 6C) after induction culture that were functional markers of hepatocytes. Notably, the AMPC cultured in this study were achieved without the addition of exogenous biochemical factors or genetic manipulation before induction.

Despite the expression of pluripotency-related characteristics in AMPC, embryonic cell surface markers SSEA-3, SSEA-4, TRA-1-60, and TRA-1-81 were not detected. A weaker expression of SSEA-1 was detected after the cell culture period, which is consistent with the acquisition of stemness, as SSEA-1 expression increased upon differentiation in human cells.^[42]^ These findings suggest that the resultant AMPC phenotype differs from that of pluripotent stem cells such as embryonic stem cells.^[43]^

## 5. Conclusions

In conclusion, our experimental results indicate that the novel method of preparing AMPC for 4 to 7 days is safe. Importantly, AMPC expresses multiple differentiation potentials, stem and self-renewal genes, and clinical autologous therapeutic potential.

## Acknowledgments

We would like to thank Wu-Chien Chien, PhD of the Department of Public Health, National Defense Medical Center, Taipei City, Taiwan for statistical consultation. We also thank Tze Chen Lim, Master for assistance with English editing. We also acknowledge the technical assistance provided by the Instrument Center of the National Defense Medical Center for various services.

## Author contributions

**Conceptualization:** Yi-Jen Lee, Jah-Yao Liu.

**Data curation:** Yi-Jen Lee.

**Funding acquisition:** Jah-Yao Liu.

**Methodology:** Yi-Jen Lee, Jehng-Kang Wang, Yu-Ming Pai, Alan Frost, Vip Viprakasit, Supachai Ekwattanakit, Hui-Chieh Chin.

**Project administration:** Jah-Yao Liu.

**Resources:** Yi-Jen Lee, Jehng-Kang Wang, Yu-Ming Pai, Alan Frost, Vip Viprakasit, Supachai Ekwattanakit, Hui-Chieh Chin, Jah-Yao Liu.

**Supervision:** Jah-Yao Liu.

**Writing – original draft:** Yi-Jen Lee.

**Writing – review & editing:** Yi-Jen Lee, Jehng-Kang Wang, Jah-Yao Liu.

## Supplementary Material
