# Supplementary material for: Culture of leukocyte-derived cells from human peripheral blood: Increased expression of pluripotent genes OCT4, NANOG, SOX2, self-renewal gene TERT and plasticity
Source: Medicine (Baltimore). 2023 Jan 20;102(3):e32746. doi: 10.1097/MD.0000000000032746 (PMC9857475; doi:10.1097/MD.0000000000032746)
Supplement: Supplementary file 1 [file medi-102-e32746-s001.pdf]

Title

Culture of leukocyte-derived cells from human peripheral blood: increased expression of pluripotent genes *OCT4, NANOG, SOX2,* self-renewal gene *TERT* and plasticity

An observational study

Authors

Yi-Jen Lee, PhD, Jehng-Kang Wang, PhD, Yu-Ming Pai, Bachelor, Alan Frost PhD, Vip Viprakasit, PhD, Supachai Ekwattanakit, PhD, Hui-Chieh Chin, Master, Jah-Yao Liu, MD, PhD*

Supplemental data 1 - Prepare alkaline dye mixture for alkaline phosphatase (ALP) staining

For ALP staining, an alkaline dye mixture was prepared by adding 40 μL of sodium nitrite solution to 40 μL of FRV-Alkaline solution and then mixed by gentle inversion. This alkaline dye mixture was allowed to stand for 2 minutes, then 1.8 mL distilled water was added to prepare diazonium salt solution. Finally, 40 μL of naphthol AS-BI alkaline solution was added to create alkaline dye mixture.
