# Supplementary material for: Culture of leukocyte-derived cells from human peripheral blood: Increased expression of pluripotent genes OCT4, NANOG, SOX2, self-renewal gene TERT and plasticity
Source: Medicine (Baltimore). 2023 Jan 20;102(3):e32746. doi: 10.1097/MD.0000000000032746 (PMC9857475; doi:10.1097/MD.0000000000032746)

Title

Culture of leukocyte-derived cells from human peripheral blood: increased expression of pluripotent genes *OCT4, NANOG, SOX2,* self-renewal gene *TERT* and plasticity

An observational study

Authors

Yi-Jen Lee, PhD, Jehng-Kang Wang, PhD, Yu-Ming Pai, Bachelor, Alan Frost PhD, Vip Viprakasit, PhD, Supachai Ekwattanakit, PhD, Hui-Chieh Chin, Master, Jah-Yao Liu, MD, PhD*

Supplemental Data 2 - Stemness genes expressions of AMPC between day-0 to day-6

(A)~(C) Stemness genes *OCT4*, *SOX2*, and *NANOG* of day-0 and AMPC (after various culture periods) of N1, N2, and N3 samples by qRT-PCR analysis. NCCIT cells were simultaneously analyzed as a positive control in every experiment. Each sample was tested in triplicate. Target gene of qRT-PCR data were normalized to a reference gene *ACTB* on each. (*T-test*, *: $p < 0.05$, **: $p < 0.005$, ***: $p < 0.0005$, ****: $p < 0.00005$, *****: $p < 0.000005$)

Supplemental Data 2 (A) Stemness genes *OCT4* of NCCIT control (Con.) and AMPC samples N1, N2, and N3 expressions.

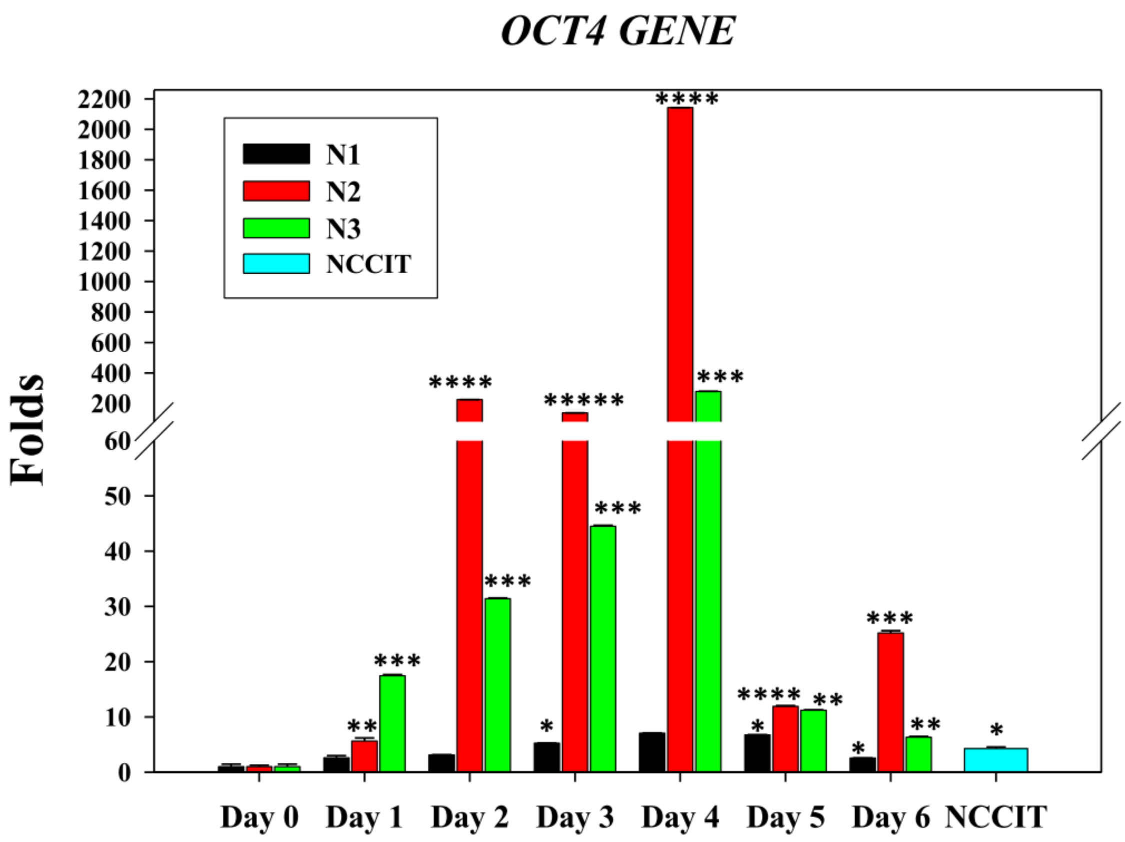

Supplemental Data 2 (B) Stemness genes *NANOG* of NCCIT control (Con.) and AMPC samples N1, N2, and N3 expressions.

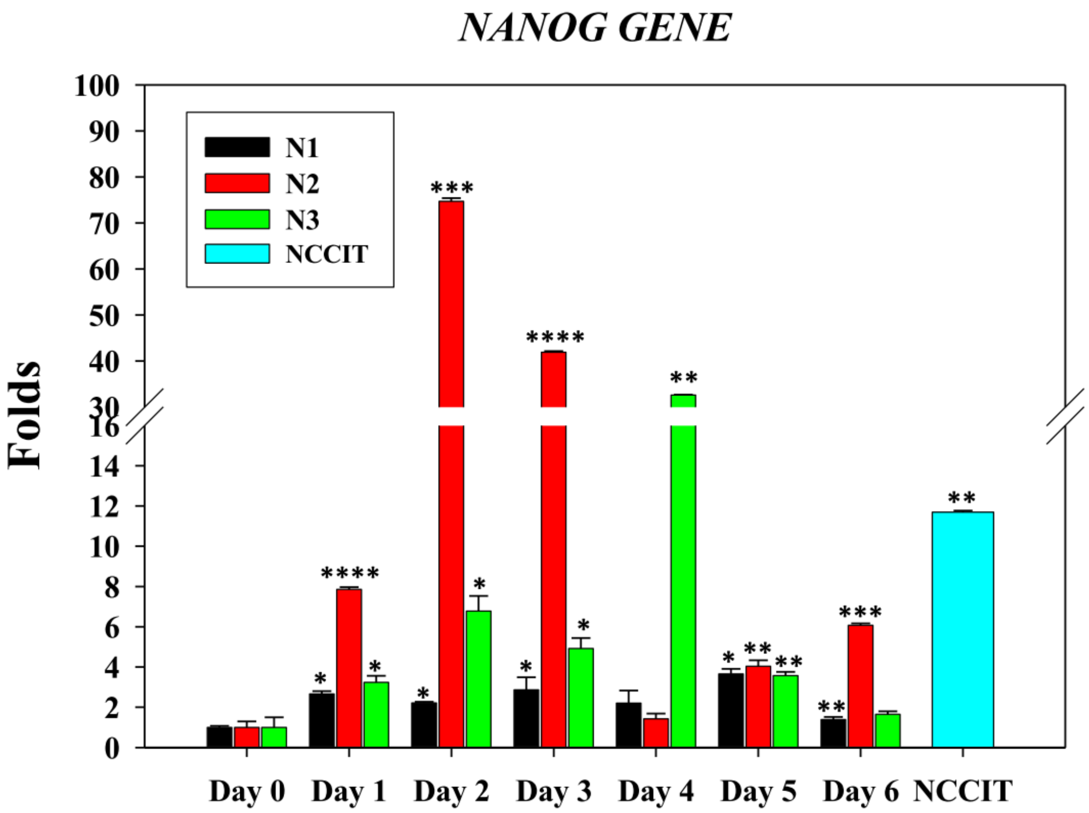

Supplemental Data 2 (C) Stemness genes *SOX-2* of NCCIT control (Con.) and AMPC samples N1, N2, and N3 expressions.

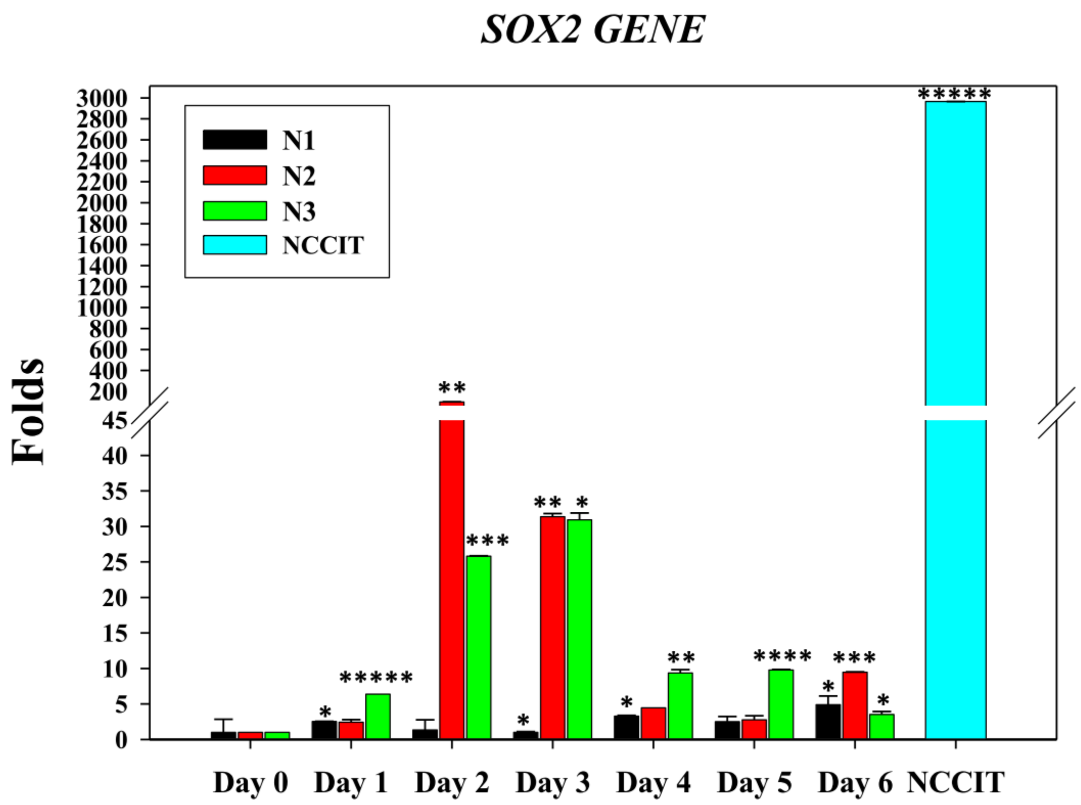

Supplemental Data 2 (D) Self-renewal gene *TERT* gene expression of day-0 and AMPC (after various culture periods) of N1, N2, and N3 samples by qRT-PCR analysis. SKOV-3 cells were simultaneously analyzed as a positive control in every experiment. Each sample was tested in triplicate. Target gene of qRT-PCR data were normalized to a reference gene *ACTB* on each. (*T-test*, * : $p < 0.05$, ** : $p < 0.005$, *** : $p < 0.0005$, **** : $p < 0.00005$)

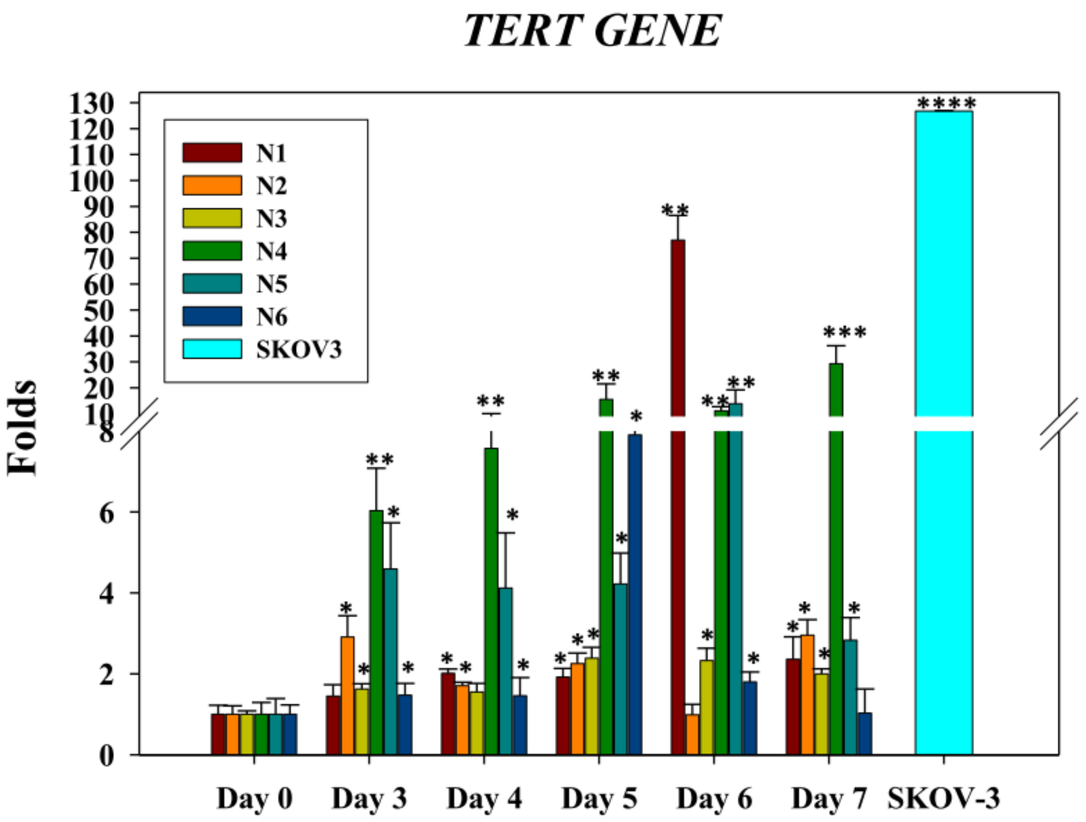

Supplement: Supplementary file 2 [file medi-102-e32746-s002.pdf]
