# Supplementary material for: Culture of leukocyte-derived cells from human peripheral blood: Increased expression of pluripotent genes OCT4, NANOG, SOX2, self-renewal gene TERT and plasticity
Source: Medicine (Baltimore). 2023 Jan 20;102(3):e32746. doi: 10.1097/MD.0000000000032746 (PMC9857475; doi:10.1097/MD.0000000000032746)
Supplement: Supplementary file 3 [file medi-102-e32746-s003.pdf]

Title

Culture of leukocyte-derived cells from human peripheral blood: increased expression of pluripotent genes *OCT4, NANOG, SOX2,* self-renewal gene *TERT* and plasticity

An observational study

Authors

Yi-Jen Lee, PhD, Jehng-Kang Wang, PhD, Yu-Ming Pai, Bachelor, Alan Frost PhD, Vip Viprakasit, PhD, Supachai Ekwattanakit, PhD, Hui-Chieh Chin, Master, Jah-Yao Liu, MD, PhD*

Supplemental information 3 - Immune-fluorescence staining of AMPC

(A) Fixation cells for immunocytochemistry (ICC) fluorescence staining. (B) Blocking of fixation cells. (C) Preparation primary antibodies and secondary antibodies. (D) Preparation ICC fluorescence-DAPI staining cells. (E) Primary and secondary antibodies (Ab).

(A) Fixation cells for immunocytochemistry (ICC) fluorescence staining

The AMPC's differentiation cells were fixed in cold methanol for 10 minutes. These fixed cells were treated with 0.1 % Triton X-100 solution for 10 minutes at room temperature, washed twice in D-PBS for 5 minutes each time to be subjected to permeabilization, following can keep on 4~10°C for 14 days until analysis.

(B) Blocking of fixation cells

Nonspecific binding sites were blocked with blocking buffer (containing 5 % bovine calf serum in 1 % bovine serum albumin solution) and then incubated for one hour at room temperature before ICC fluorescence staining.

(C) Preparation primary antibodies and secondary antibodies

Primary antibodies were diluted in 1 % bovine serum albumin solution. These AMPC's differentiation permeabilized cells were incubated with the primary antibody overnight at 4°C cold room, then washed for 5 minutes in D-PBS. The secondary antibodies were diluted in 1 % bovine serum albumin solution. Cells were incubated with the secondary antibodies for 1 hour at room temperature in the dark, washed for 5 minutes in D-PBS.

(D) Preparation ICC fluorescence-DAPI staining cells

The 4', 6-diamidinno-2'-phenylindole, dihydrochloride (DAPI, Thermo) stock solution was diluted by 1:3000 in D-PBS solution. The ICC fluorescence staining cells also were incubated with the DAPI solution for 5 minutes at room temperature in the dark, and then washed for 5 minutes in D-PBS, named ICC fluorescence-DAPI staining cells.

(E) Primary and secondary antibodies (Ab)

Anti-human Ab	Brand	Primary Ab host	Primary Ab dilution	Secondary Ab dilution
Nestin	Millipore (MAB5326)	Mouse monoclonal	1:100	1:500*2
Neurogenin 3	Santa Cruz (sc-376607)	Mouse monoclonal	1:50	1:500*2
Myogenin	Millipore (MAB3876)	Mouse monoclonal	1:200	1:500*2
Troponin I	Abnova (H00007137-M04)	Mouse monoclonal	10 μg/ml	1:500*1
Alpha-actinin	Millipore (05-384)	Mouse monoclonal	30 μg/ml	1:500*2
Connexin 32	e-Bioscience (14-9759)	Mouse monoclonal	30 μg/ml	1:500*2
Cytochrome P450 (CYP1A1)	Santa Cruz (sc-25304)	Mouse monoclonal	1:50	1:500*2
Albumin	Sigma (A6684)	Mouse monoclonal	1:500;	1:500*2

Note:

*1: FITC-anti-Rabbit IgG monoclonal was obtained by BioLegend (406403).

*2: FITC-anti-Mouse IgG monoclonal was obtained by abcam (ab6785).
